# Morphometric analysis of rat femoral vessels under a video magnification system

**DOI:** 10.1590/1677-5449.011016

**Published:** 2017

**Authors:** Rui Sergio Monteiro de Barros, Rafael Aquino Leal, Renan Kleber Costa Teixeira, Vitor Nagai Yamaki, Felipe Lobato da Silva Costa, Daniel Haber Feijó, Andre Lopes Valente

**Affiliations:** 1 Universidade do Estado do Pará – UEPA, Belém, PA, Brazil.

**Keywords:** rats, femoral vein, femoral artery, anatomy, ratos, veia femoral, artéria femoral, anatomia

## Abstract

The right femoral vessels of 80 rats were identified and dissected. External lengths and diameters of femoral arteries and femoral veins were measured using either a microscope or a video magnification system. Findings were correlated to animals’ weights. Mean length was 14.33 mm for both femoral arteries and femoral veins, mean diameter of arteries was 0.65 mm and diameter of veins was 0.81 mm. In our sample, rats’ body weights were only correlated with the diameter of their femoral veins.

## Resumo

Os vasos femorais de 80 ratos foram identificados e dissecados. O comprimento e o diâmetro externo da artéria e da veia femoral foram mensurados com o auxílio de um microscópio e um sistema de magnificação por vídeo. Esses parâmetros foram correlacionados com o peso do animal. O comprimento de ambos os vasos femorais foi de 14,33 mm, e a média do diâmetro das artérias foi de 0,65 mm, e das veias, de 0,81 mm. Na nossa amostra, o peso dos animais se correlacionou apenas com o diâmetro da veia femoral.

## INTRODUCTION

Anatomic knowledge is extremely important when using animals to reproduce clinical situations for validation of experimental models of diseases and surgical conditions.^1^^,^ 2 The femoral vessels of rats are an ideal resource for beginners to practice microvascular surgery,^1^^,^ 3 ^,^ 4 since they resemble the human digital arteries and veins,^5^^,^ 6 and they are universally accepted for practicing microsurgery skills.^1^^,^ 3 ^,^ 4 However, normally only adult rats are used for microanastomosis, because their femoral vessels are of suitable size. Tiny vessels make safe manipulation, adventitia removal, vessel lumen dilation, and final anastomosis difficult.^7^

Knowledge of the anatomic features of rat femoral vessels is essential for microsurgery training and research, but there are scant data in the literature on morphometric analysis of rat femoral vessels.^8^ Therefore, there is a need to quantify their length and diameter and correlate these measurements to the animals’ weights. However, while microscopes offer excellent resolution, correctly measuring the external morphometric parameters of femoral vessels is very difficult.^2^^,^ 5 ^,^ 8

In this scenario, video microsurgery comes into its own because video systems using digital technology, with camcorders to capture images and transmit them in real time to HD TV sets, offer surprisingly high quality, good enough to enable precise and safe manipulation of delicate tissues.^9^^-^ 13 Magnification with video systems^12^^,^ 13 is low cost and high resolution and also offers the possibilities of recording and sharing images, simplifying measurement of tiny vessels and nerves.

To date, video systems have only been used for arterial microanastomosis.^10^^-^ 13 No studies have evaluated this magnification system for vein or nerve repair or for training. It is therefore necessary to describe the anatomic features of rat femoral vessels must to facilitate training using rats and for future studies. The present study aims to evaluate the external morphometric parameters of Wistar rat femoral vessels with the assistance of a video magnification system, to compare the results to using a microscope, and to establish relationships between measurements and animals’ weights.

## METHODS

The study followed the rules set out in the Brazilian Law for Animal Care (Law: 11.794/08) and the project was approved in advance by the Animal Use and Care Committee at the Universidade do Estado do Pará (UEPA) (protocol 13/09). Forty male and 40 female Wistar rats (*Rattus norvegicus*) were obtained from the Animal House at the Experimental Surgery Laboratory, Universidade do Estado do Pará (UEPA), and kept in a controlled environment with food and water *ad libitum*.

The animals were randomly assigned to two groups (20 males and 20 females per group): either a microscope group, in which microsurgical procedures were performed under a DFVasconcelos^©^ microscope with image magnification of 40×, or a video system group,^12^^,^ 13 in which microsurgical procedures were performed with the aid of a video system comprising a high-definition Sony^©^ Handycam HDR-XR160 set to 24× magnification, macro lenses, a high-definition, 42-inch, LED television, and a digital HDMI cable. The video camera was set vertically 9.3 cm above the surgical field, held by a reversed base tripod. Two low-intensity halogen light sources were used near to the camera to provide adequate illumination of the surgical field.

All surgical procedures were performed by the same surgeon. All animals underwent the same surgical procedure, with the two groups differing only in the magnification technique used. The rats were anesthetized with intraperitoneal ketamine (70 mg/kg) and xylazine (10 mg/kg) and then shaved and placed in a horizontal supine position. Antisepsis was performed for all right hind legs. Under direct view, a longitudinal incision was made in the inguinal ligament, followed by blunt dissection between subcutaneous tissues to expose the femoral neurovascular bundle.

**Figure 1:**
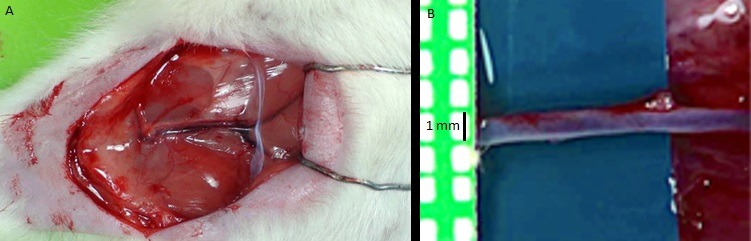
Photographs of femoral vessels: A) Macroscopic view; B) Femoral vein under video system magnification.

The femoral vessels were identified and individualized. A blue rubber shield was used for better visualization of vessels. Lidocaine 2% was used to prevent spasm. The limits of femoral vessels^8^ were defined from the inguinal ligament to the superficial epigastric branch. External morphometric parameters were measured with the aid of a millimeter ruler (Figure 1) and confirmed by digital caliper. After these data had been collected, the specimens were used by for microsurgical training.

Prior to analyses, Levene’s test and the Kolmogorov-Smirnov test were used to test for homogeneity of variances and normality of response variables. Student’s *t* test was used to test for relationships between animals’ sex and diameters and lengths of vessels. The linear correlation test was used to test for relationships between diameters and lengths of vessels and animals’ weights. A significance level of 5% was adopted as cutoff for rejection of the null hypothesis.

## RESULTS

In all animals studied, only one right femoral artery and vein were found per animal, there was one branch (superficial circumflex iliac branch) located at the proximal third of the arterial graft and the middle of the venous vessel. The lengths of femoral arteries and femoral veins were identical and there were no statistically significant difference between the groups (microscope: 14.44 ± 1.88 mm vs. video system: 14.22 ± 1.76 mm; p = 0.82). There was no statistically significant difference between sexes in terms of vessel length using either magnification technique.

There were also no significant differences between groups for diameters of femoral arteries (microscope: 0.64 ± 0.16 mm vs. video system 0.66 ± 0.14 mm; p = 0.91) or of femoral veins (microscope: 0.84 ± 0.30 mm vs. video system 0.78 ± 0.22 mm; p = 0.76). There was no statistically significant relationship between animals’ sex and either diameter or length of femoral artery or vein in either group.

The mean weight of the animals was 308.28 ± 32.17 g in the microscope group and 297.54 ± 29.44 g in the video group. No statistical relationship was found between length of either vessel and artery diameter, but there was a positive relationship between animals’ weights and vein diameters in both the microscope [r (Pearson): 0.80; p = 0.0242] and video groups [r (Pearson): 0.82; p = 0.0156].

## DISCUSSION

Video microsurgery offers several advantages,^12^^-^ 14 such as: (1) better ergonomics; (2) access to the patient without interposition of microscope equipment; (3) easy assembly; (4) portability; (5) lower cost; (6) easy image recording; (7) increased surgical team involvement and commitment; and (8) high-definition imaging of tiny structures. The main limitation of the video-assisted system is the lack of a stereoscopic view, although, as with laparoscopic and endoscopic surgery, information on spatial depth can be inferred from secondary spatial depth cues and from experience.

Sex did not influence external morphometric parameters, allowing both sexes to be used for surgery practice. Normally, female animals are less often used for research,^15^ so they can be used for training, reducing costs for the laboratory and animal house. Usually, fatter animals are used for training because it is believed that they have larger vessels,^3^^,^ 8 ^,^ 16 but in our data only venous diameter was affected by this parameter. This demonstrates that there is little advantage from using fatter rats, since venous anastomoses are difficult to construct because of thinner walls.

The diameters and lengths of the femoral vessels studied were similar to those observed by other authors for these vessels,^2^^,^ 7 providing additional evidence that femoral vessels are a good model for microsurgery training. However, this model does have certain limitations, such as the absence of venous valves^2^ and thinner walls than human vessels.^8^

There were no differences between the two groups in any of the characteristics assessed, showing the great potential that video systems offer as a magnification technique for training and for future studies, particularly since the cost of our proposed magnification system is likely to be less than US$ 2,500, in contrast with the US$ 20,000 price of the microscope.^9^^-^ 12 Therefore, using such a video system is a feasible alternative for practicing microsurgery at low cost and it is also a portable method that can be easily made available, allowing it to be potentially included in residency programs.^7^

We believe there is still scope to improve this proposal. Refinements can be made in two areas: improving the surgeon’s skill with experimental video system magnification training^12^ and acquiring an imaging system offering higher resolution, which could also include use of three-dimensional video cameras.^14^

In summary, on the basis of the methodology used in this study, we conclude that the average length of both femoral vessels was 14.33 mm, the mean diameter of arteries was 0.65 mm, and mean vein diameter was 0.81. In our sample, rats’ weights were only correlated to the diameter of their femoral veins. There were no statistically significant differences between the results of using a microscope or video system magnification.

## References

[1] Yen DM, Arroyo R, Berezniak R, Partington MT (1995). New model for microsurgical training and skills maintenance. Microsurgery.

[2] Teixeira RK, Yamaki VN, Valente AL (2015). Do the femoral veins of female Wistar rats have valves?. J Vasc Bras.

[3] Chan WY, Matteucci P, Southern SJ (2007). Validation of microsurgical models in microsurgery training and competence: a review. Microsurgery.

[4] Balasundaram I, Aggarwal R, Darzi LA (2010). Development of training curriculum for microsurgery. Br J Oral Maxillofac Surg.

[5] Sucher R, Oberhuber R, Rumberg G (2010). A rapid vascular anastomosis technique for hind-limb transplantation in rats. Plast Reconstr Surg.

[6] Sucher R, Oberhuber R, Margreiter C (2010). Orthotopic hind-limb transplantation in rats. J Vis Exp.

[7] Lima DA, Galvão MS, Cardoso MM, Leal PR (2012). Laboratory training program in microsurgery at the National Cancer Institute. Rev Bras Cir Plast..

[8] Blain B, Zhang F, Jones M (2001). Vascular grafts in the rat model: an anatomic study. Microsurgery.

[9] El-Shazly M, El-Sonbaty M, Kamel A, Zaki M, Frick A, Baumeister R (2003). Endoscopic-assisted microsurgery: microsurgery in the new millennium? A comparative experimental study. Br J Plast Surg.

[10] Gorman PJ, Mackay DR, Kutz RH, Banducci DR, Haluck RS (2001). Video microsurgery: evaluation of standard laparoscopic equipment for the practice of microsurgery. Plast Reconstr Surg.

[11] Nissen NN, Menon VG, Colquhoun SD, Williams J, Berci G (2013). Universal multifunctional HD video system foi minimally invasive open and microsurgery. Surg Endosc.

[12] Barros RS, Brito MV, Moura GP (2011). Is it possible to do a microvascular anastomosis with an ordinary video camera? Experimental study. J Reconstr Microsurg.

[13] Wong AK, Davis GB, Nguyen TJ (2014). Assessment of three-dimensional high-definition visualization technology to perform microvascular anastomosis. J Plast Reconstr Aesthet Surg.

[14] Liu J, Chen B, Ni Y, Zhan Y, Gao H (2014). Application of a three-dimensional microsurgical video system for a rat femoral vessel anastomosis. Chin Med J (Engl).

[15] Zucker I, Beery AK (2010). Males still dominate animal studies. Nature.

[16] Hölzen JP, Palmes D, Langer M, Spiegel HU (2005). Microsurgical training curriculum for learning kidney and liver transplantation in the rat. Microsurgery.

